# MicroRNA expression profiles in avian haemopoietic cells

**DOI:** 10.3389/fgene.2013.00153

**Published:** 2013-08-14

**Authors:** Yongxiu Yao, Jane Charlesworth, Venugopal Nair, Mick Watson

**Affiliations:** ^1^Avian Viral Diseases Programme, Compton Laboratory, The Pirbright InstituteBerkshire, UK; ^2^ARK-Genomics, Royal (Dick) School of Veterinary Studies, The Roslin Institute, University of EdinburghEdinburgh, UK

**Keywords:** microRNA, B-cell, macrophages, DT40, HD11, IAH30

## Abstract

MicroRNAs (miRNAs) are small, abundant, non-coding RNAs that modulate gene expression by interfering with translation or stability of mRNA transcripts in a sequence-specific manner. A total of 734 precursor and 996 mature miRNAs have so far been identified in the chicken genome. A number of these miRNAs are expressed in a cell type-specific manner, and understanding their function requires detailed examination of their expression in different cell types. We carried out deep sequencing of small RNA populations isolated from stimulated or transformed avian haemopoietic cell lines to determine the changes in the expression profiles of these important regulatory molecules during these biological events. There were significant changes in the expression of a number of miRNAs, including miR-155, in chicken B cells stimulated with CD40 ligand. Similarly, avian leukosis virus (ALV)-transformed DT40 cells also showed changes in miRNA expression in relation to the naïve cells. Embryonic stem cell line BP25 demonstrated a distinct cluster of upregulated miRNAs, many of which were shown previously to be involved in embryonic stem cell development. Finally, chicken macrophage cell line HD11 showed changes in miRNA profiles, some of which are thought to be related to the transformation by v-*myc* transduced by the virus. This work represents the first publication of a catalog of microRNA expression in a range of important avian cells and provides insights into the potential roles of miRNAs in the hematopoietic lineages of cells in a model non-mammalian species.

## Introduction

MicroRNAs (miRNAs) are a large class of endogenous non-coding RNAs 21–23 nucleotides in length. Since they were first described nearly 20 years ago, there has been a steady increase in their discovery and the latest Release 20 of miRBase has 24,521 entries of miRNAs from various species, including 734 mature miRNAs from *Gallus gallus* (www.mirbase.org). Many of these miRNAs are expressed differentially during development in a cell-specific manner. A number of previous studies in mammals have demonstrated hematopoietic lineage-specific expression of miRNAs, suggesting key roles for these molecules in controlling hematopoietic machinery (Ramkissoon et al., 2006; Merkerova et al., 2008). Chickens are used as a model organism for a number of studies and the developing chicken embryo has been shown to be an excellent biological system to study the repertoire and dynamics of small regulatory RNAs (Glazov et al., 2008). The development of deep sequencing technologies and bioinformatics pipelines has greatly facilitated the discovery and quantification of expression levels of miRNAs in different cell types (Friedlander et al., 2008). We and others have previously reported the expression profiles of miRNAs in chicken T cells transformed by Marek's disease virus (MDV), and shown that the majority of the miRNAs expressed in these cell types are of viral origin (Burnside et al., 2008; Yao et al., 2009; Morgan and Burnside, 2011). Elevated expression of miRNAs such as gga-miR-155 has also been demonstrated in chicken hematopoietic cells transformed by reticuloendotheliosis virus (Bolisetty et al., 2009). However, studies examining the global expression of miRNAs in different haemopoietic cell lineages in chickens have not yet been carried out.

In this study we carried out deep sequencing of the miRNAs of six avian haemopoietic cell populations: **BP25**, a chick embryonic stem cell (cESC) line; **Bu1B**, naïve embryonic B lymphocytes; **StimB**, CD40L-induced B-cells; **DT40**, an avian-leukosis virus (ALV) transformed B-cell line; **HD11**, a chicken macrophage cell line; and **IAH30**, a turkey macrophage cell line. We have determined the miRNA expression profile of the cESC line, and we have compared the miRNA expression profiles of naïve B cells with B-cells after stimulation with the CD40 ligand (CD40L), to gain an understanding of the global changes in miRNA expression after signaling through the CD40 ligand interaction. We have also determined the miRNA profiles in the ALV-transformed B-cell line DT40 (Bachl et al., 2007) to identify the effects of the c-*myc*-induced transformation on miRNA expression. To further examine the effects of *myc*-induced transformation on other cell types of the haemopoietic lineage, we have determined the miRNA expression profiles of the chicken macrophage cell line HD11 (Beug et al., 1979). In addition, we also examined the miRNAs in the turkey macrophage cell line IAH30 (Lawson et al., 2001) to compare the miRNA profiles of chicken and turkey macrophages.

By analyzing the changes in expression of miRNA populations in the different cell types, our study provides insights into the cell type-specific miRNA signatures of avian haemopoietic cells. We believe that our data will be helpful in identifying targets and pathways associated with a number of phenotypic and functional characteristics of these lineages of avian haemopoietic cells.

## Materials and methods

We made use of six cell lines for the characterization of the miRNAs: BP25 cESC line was propagated on irradiated STO feeder cells (ATCC collection) as previously described (Pain et al., 1996). Naïve B cell population was prepared from embryonic bursa of Fabricius (BF) or spleen collected from line 0 eggs at 18-day-old of embryonation. Briefly, BF was dissected from embryos and cell suspensions were separated by density gradient centrifugation on Ficoll-Paque under sterile conditions. B-cell preparations of more than 95% purity were obtained by magnetic cell sorting on MACS separation columns LS (Miltenyi Biotec, UK) using chicken B-cell (Bu-1)-specific monoclonal antibody AV20 (Rothwell et al., 1996) and anti-mouse microbeads as previously described (Kothlow et al., 2008). CD40L-induced *in vitro* B-cell proliferation was carried out as previously described using purified recombinant protein (Tregaskes et al., 2005; Kothlow et al., 2008), and cells were harvested 48 h after treatment with the ligand. DT40 (Buerstedde et al., 2002; Bachl et al., 2007), HD11 (Beug et al., 1979) and IAH30 (Lawson et al., 2001) cell lines were propagated as previously described.

RNA extraction for miRNA profiling was carried out as previously described (Yao et al., 2012) using miRVana miRNA isolation kit (Ambion, UK). Sequencing of the miRNAs was carried out on the Illumina GAIIx and 36 base-pair single-end sequencing. After sequencing, adaptor and primer/dimer sequences were removed using Cutadapt (http://code.google.com/p/cutadapt/). Using the Novoalign short read aligner (www.novocraft.com), we mapped the reads from all the individual cell lines, including the turkey macrophage cell line, to the known chicken mature miRNAs downloaded from miRBase (www.miRBase.org) version 19. Reads mapping to each miRNA were counted and used as input for downstream analyzes. To correct for differences in library size and sequencing depth, raw mapped read counts were scaled to reads per million mapped reads (Mortazavi et al., 2008). Changes in miRNA expression in CD40L-stimulated (StimB) cells compared to naïve B cells (Bu1B) were calculated as log2 ratios of normalized (RPM) counts. Similarly, changes in the expression of individual miRNAs in DT40 cells were also calculated in comparison to those of naïve B cells (BU1B). Normalized counts of miRNA levels were used to generate a heatmap in order to identify candidate miRNAs that are differentially expressed in different cell lines. The pearson correlation coefficient was used as a similarity measure in the heatmap cluster analyzis, and using the “average” agglomeration method. Validation of expression levels of gga-miR-21, gga-miR-26a, gga-miR142-3p, gga-miR-155 and gga-miR-223 was carried out by quantitative RT-PCR using procedures described (Yao et al., 2008).

## Results

### Raw data

The raw data hava been submitted to the European Nucleotide Archive (ENA) under accession number ERP002558. Counts and normalized RPM values have been uploaded as Supplementary Material.

### miRNA expression in B-lymphocytes after CD40L stimulation

**Figure 1:**
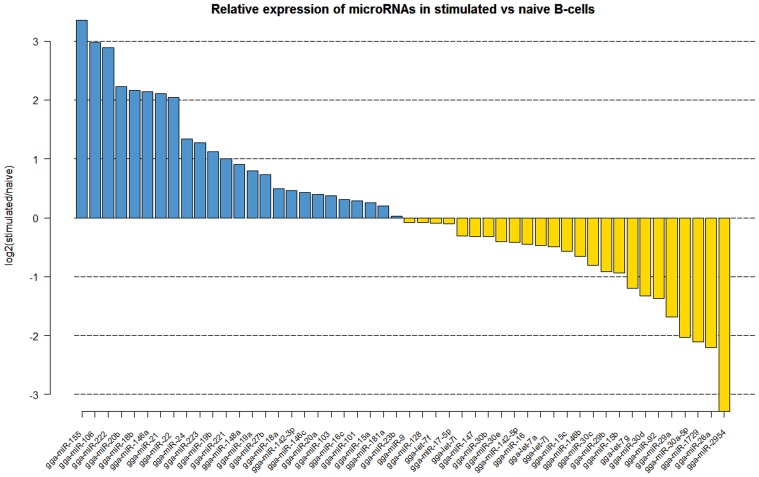
**Log2 fold (stimulated/naïve) change in expression (red or green bars indicating increased or decreased expression respectively) of the 50 most abundantly expressed miRNAs in CD40L-stimulated B cells (StimB) compared to the naïve B cells (BU1B)**.

Naïve B cells are activated through a combination of signals from the antigen and through the binding of the CD40 ligand to CD40 which drives proliferation. Comparison of the naïve and CD40L-stimulated B cells revealed significant changes in the miRNA expression profiles (Figure 1). The miRNAs which showed significant increase upon CD40L-stimulation included gga-miR-21, gga-miR-155, gga-miR-146a, gga-miR-20b, gga-miR-106, gga-miR-222, and gga-miR-22. A number of miRNAs were also downregulated after CD40L stimulation. This included gga-miR-26a which showed a 4.5-fold decrease in expression. Other downregulated miRNAs included members of the miR-30 family of miRNAs (gga-miR-30c, gga-miR-30d, gga-miR-30a-5p) and the avian-specific gga-miR-1729 originally discovered in developing chick embryo.

### miRNA expression in DT40 cells

**Figure 2:**
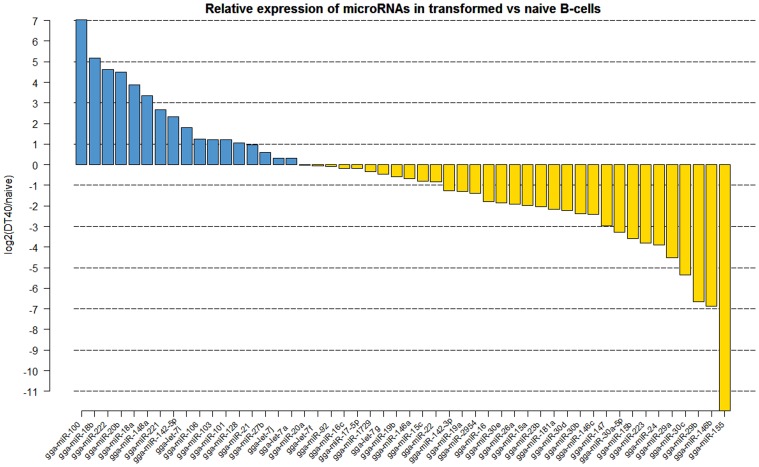
**Log2 fold (DT40/naïve) change in expression (red or green bars indicating increased or decreased expression respectively) of the 50 most abundantly expressed miRNAs in DT40 ALV-transformed B cells (DT40) compared to the naïve B cells (BU1B)**.

**Figure 3:**
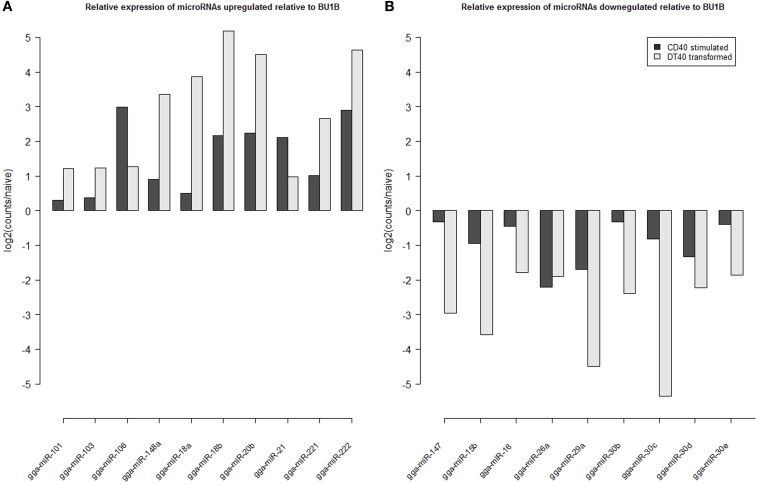
**Log2 fold change in expression of microRNAs (A) up-regulated in CD40L stimulated (StimB) and DT40 transformed cells (DT40) relative to naïve B cells (BU1B); and (B) down-regulation in CD40L stimulated (StimB) and DT40 transformed cells (DT40) relative to naïve B cells (BU1B)**.

Analyzis of the expression of miRNAs in DT40 cells demonstrated overexpression of a number of miRNAs compared to the naïve Bu1B positive cells (Figure 2). Among the upregulated miRNAs in DT40 cells, many miRNAs including gga-miR-18a and -18b, -222, -20b, -148a, -221, -106, -103, -101 and -21 also increased in CD40L-stimulated cells (Figure 3A). On the other hand, gga-miR-100 is highly upregulated in DT40 cells compared to naïve B cells or CD40L-stimulated cells (data not shown). Similarly, gga-miR-146a, upregulated in CD40L-stimulated B cells, was down-regulated in DT40 cells, providing further evidence for its role in the immune system. A number of miRNAs, including gga-miR-16, -30e, -30d, -30b, -30c, -26a, -147, -15b, and -29a, down-regulated in DT40 cells were also downregulated in CD40L-treated cells (Figure 3B). The most striking change was in the level of gga-miR-155. This miRNA was the most up-regulated miRNA in CD40L-stimulated B cells, but was downregulated in the DT40 cells.

**Figure 4:**
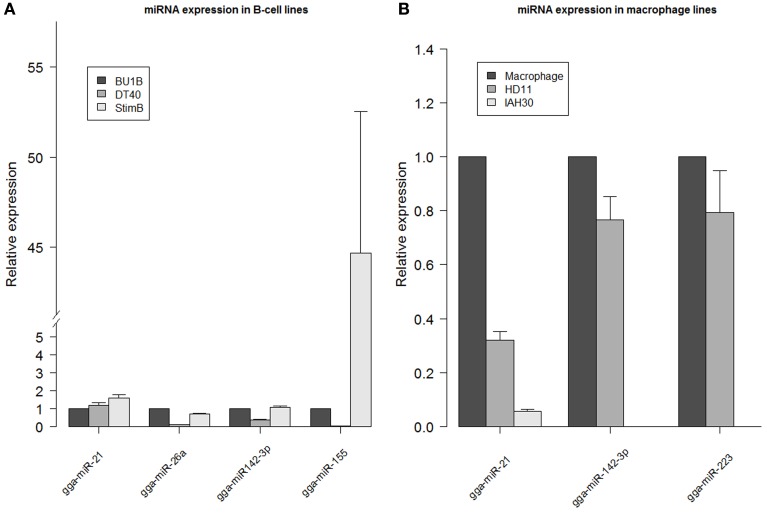
**miRNA expression levels determined by qRT-PCR**. Relative expression of gga-miR-21, gga-miR-26a, gga-miR142-3p and gga-miR-155 measured in RNA extracted from DT40 and StimB cells compared to Bu1B cells **(A)** and gga-miR142-3p and gga-miR-223 in HD11 and IAH30 compared to chicken macrophages **(B)**. Results represent the mean of triplicate assays with error bars showing the standard error of the mean. Data normalized to gga-let-7a. Note the broken axis in **(A)**.

Expression levels of miRNAs obtained from the deep sequencing data were validated by carrying out quantitative RT-PCR on gga-miR-21, gga-miR-26a, gga-miR-142-3p and gga-miR-155 using RNA extracted from different cell types. Differences in the expression profiles of the miRNAs determined from deep sequencing broadly agreed with the quantitative RT-PCR data from naïve Bu1B-positive, DT40 and stimulated B cells (Figure 4A). Similarly, the high expression levels of gga-miR-142-3p and gga-miR-223 in HD11 cells was also confirmed by quantitative RT-PCR (Figure 4B).

### miRNA expression in avian macrophage cell lines HD11 and IAH30

Out of the 718,959 sequences in HD11 cells that mapped to the mature miRNAs in miRBase, the highest level of expression was observed for gga-miR-21, which accounted for 28.8% of all miRNAs expressed in these cells. The high level of gga-miR-21 was demonstrated in normal macrophages also (Figure 4B). Other miRNAs which are expressed at high levels in HD11 include gga-miR-142-3p (10.5%), gga-miR-223 (6.9%), gga-miR-19b (4%), gga-miR-20a (3.7%) and gga-miR-22 (3.4%).

More than half (53%) of the total 175,408,236 sequences from the IAH30 turkey cell line mapped to known mature chicken miRNA sequences. Of those, a large majority matched with the gga-miR-21 (33.4%). Other chicken miRNAs that are expressed at high levels in IAH30 cells include gga-miR-24 (5.5%), gga-miR-27b (4%), gga-miR-19b (3.9%), gga-miR-20a (3.9%), gga-miR-148a (3.7%), gga-miR-23b (3%), and gga-miR-92 (3%). Further studies are required to identify the functional significance of these miRNAs and further characterize the other turkey miRNAs. Interestingly, gga-miR-142-3p and gga-miR-223 were significantly downregulated in IAH30 cells compared to HD11 cells Figure 4 For gga-miR-142-3p, there are only 19 reads in IAH30 compared to 75,670 reads in HD11. Similarly, there are 10 and 50,275 reads representing gga-miR-223 in IAH30 and HD11 respectively. This difference is further confirmed by qRT-PCR (Figure 4B).

### miRNA expression in cESC line BP25

Sequencing from cell line BP25 gave a total of 1,624,435 reads out of which 1,146,503 (70.6%) passed QC and mapped to known mature chicken miRNAs. The most predominant miRNA population expressed in BP25 was indeed the ES cell-specific miRNAs belonging to the miR-302-367 cluster. The expression levels of five miRNAs in the miR-302-367 cluster (miR-302a, miR-302b, miR-302c, miR-302d, and miR-367) accounted for 39.5% of all the sequenced miRNAs. Another miRNA expressed at high levels in the BP25 cell line is gga-miR-21 that accounted for 23.3% of the miRNAome. In addition, miR-17-92 cluster of miRNAs (miR-17, miR-18a, miR-19a, miR-20a, miR-19b-1, and miR-92-1) was also expressed at relatively high levels, accounting for 12.4% of the miRNAome in BP25.

### Comparison of the miRNA profiles of different avian cell types

**Figure 5:**
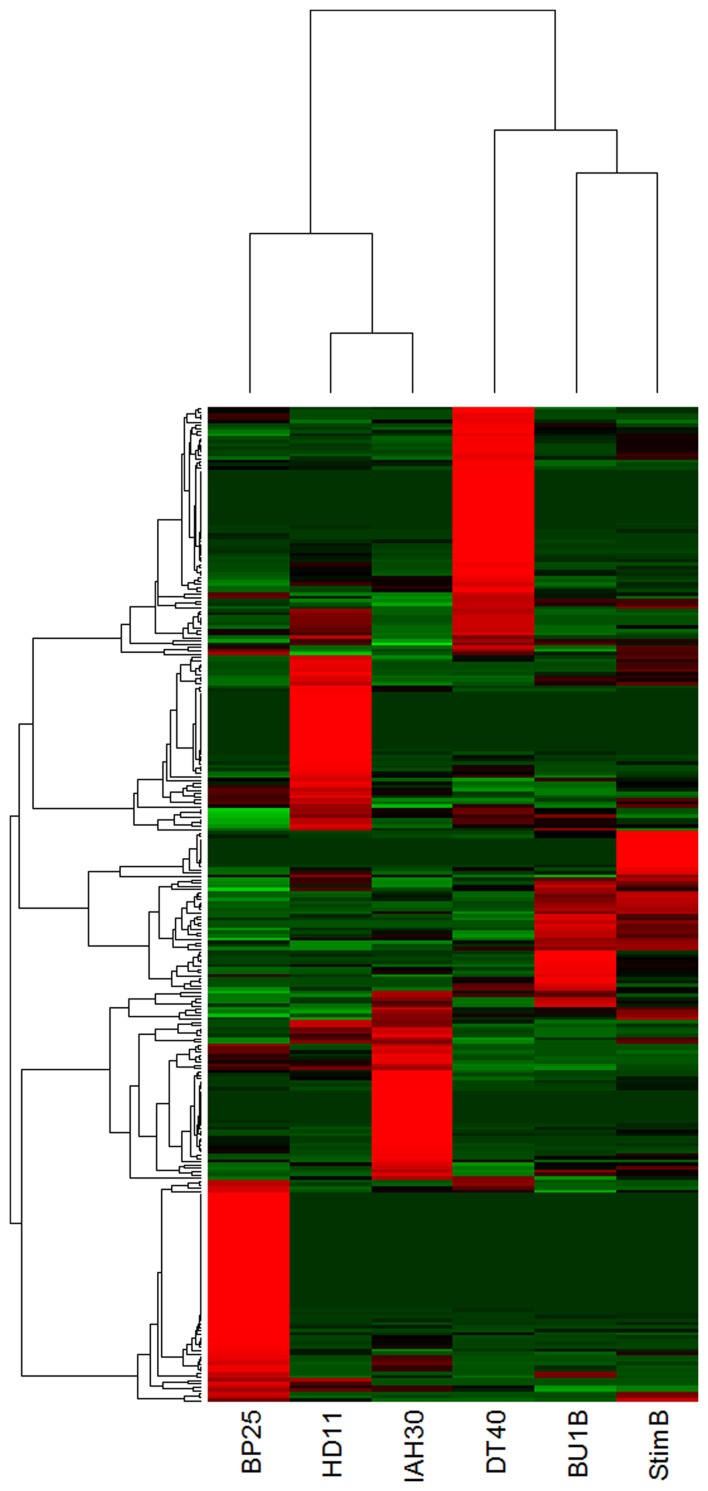
**Heatmap demonstrating differential clustering of the miRNAs from avian cell types based on expression levels calculated from normalized read counts (Red indicates high levels of expression and green to indicate low levels)**. BP25 is the cESC line BP25 (Pain et al., 1996); BU1B is the naïve embryonic B lymphocyte; DT40 is the data from DT40 cells (Buerstedde et al., 2002); stimB is the CD40L-induced B-cells; HD11 and IAH30 represent HD11 (Beug et al., 1979) and IAH30 (Lawson et al., 2001) cell lines respectively.

Comparison of the differential expression of miRNA, based on the normalized counts, showed clustering of miRNAs in various avian cell types (Figure 5). For example, cESC line BP25 clearly demonstrated clustering of highly expressed ES cell-specific miRNAs, which are not expressed in any of the other cell lines. Similarly, DT40 cells showed a distinct profile of miRNA expression. The two cell lines IAH30 and HD11 coming from different species (turkey and chicken respectively), clearly showed distinct expression profiles despite being of macrophage origin. Naïve and stimulated B cells showed clustering based on miRNA expression profiles, yet demonstrated specific miRNA expression patterns, with a group of miRNAs showing high expression in stimulated B cells that are not present in the other cell lines.

## Discussion

Deep sequencing using Illumina platform can be valuable for obtaining miRNAome data, and we have used this for determining the miRNA expression levels from cell lines of chicken, a model avian species. Comparison of the normalized read counts was used to obtain digital data on expression levels of individual, already known mature miRNAs.

As in mammals, B-lymphocytes in birds are one of the critical components of the immune system responsible for the production of antibodies to specific antigens, pathogens and vaccines. Chickens have a distinct organ called BF in which the naïve B cells mature before being exported to the periphery. Naïve B cells are activated through a combination of signals from the antigen and through the binding of the CD40 ligand to CD40 which drives proliferation. The miRNAs which showed significant increase upon CD40L-stimulation included gga-miR-21, gga-miR-155, gga-miR-146a, gga-miR-20b, gga-miR-106, gga-miR-222 and gga-miR-22 (Figure 2). Some of these miRNAs have already been well-documented for their roles in cell proliferation and cancer. The highly expressed gga-miR-155 has been extensively studied and shown to be associated with cell proliferation in a number of cancers, as well as in autoimmunity (Leng et al., 2011; Wang and Wu, 2012). Interestingly, miR-155 was first discovered in the chicken as part of the c-*bic* transcript in ALV-transformed lymphomas (Clurman and Hayward, 1989). High expression of miR-155 upon CD40L stimulation is consistent the major role of this miRNA in proliferation. Other viral oncogenes such as v-Rel have also been shown to drive miR-155 expression (Bolisetty et al., 2009). As v-Rel is an NF-κ B homolog, it is possible that the increased expression of miR-155 by CD40L is mediated through the NF-κ B pathway, although other signaling systems may also be involved. Another miRNA that shows increase in expression after CD40L-stimulated B-cells is gga-miR-146a. As a multifaceted miRNA, its role in hematopoiesis, immune response and cancer has been well documented (Labbaye and Testa, 2012). Activation of miR-146a, thought to be through the NF-κ B pathway, has also shown to be important in the innate immune responses (Williams et al., 2008). Our study demonstrating the upregulation of miR-146a through CD40L interaction further adds to our understanding of the molecular pathways of biogenesis of miR-146a and CD40L functions.

A number of miRNAs were also down-regulated after CD40L stimulation. This included gga-miR-26a which showed a 4.5-fold decrease in expression. Interleukin-2 (IL-2) is essential for the growth and proliferation of T-cells (Cantrell and Smith, 1984), and we have previously shown the downregulation of miR-26a in MDV-transformed cell lines, where the decreased expression relieved its suppressive effect on the interleukin-2, potentially allowing proliferation (Xu et al., 2010). It is possible that the CD40L-stimulation also makes use of a similar pathway. The most down-regulated miRNA, which decreased in expression by almost 10-fold, was gga-miR-2954. This miRNA has only been reported in birds (*Gallus gallus* and *Taeniopygia guttata*) and was originally identified as being male-specific during early chick development (Zhao et al., 2010). Another avian-specific miRNA, gga-miR-1729 was also down-regulated, a miRNA originally discovered in developing chick embryo (Glazov et al., 2008). The change in expression after CD40L stimulation suggests that these miRNAs have roles beyond early chick development. Other down-regulated miRNAs include members of the miR-30 family of miRNAs (gga-miR-30c, gga-miR-30d, gga-miR-30a-5p) many of which have been implicated in a wide range of cancers (Gaziel-Sovran et al., 2011; Baraniskin et al., 2012; Cheng et al., 2012). Suppression of these miRNAs may therefore be related to cell division and proliferation of the B cells after stimulation with CD40L. By comparing the miRNA expression in naïve and CD40L-activated B-cells, we were able to identify changes in miRNA expression related to the CD40L-induced proliferation.

ALV-transformed cell line DT40 is extensively used in molecular genetic studies because it has high levels of recombination, making it a very useful system for *in vitro* gene knock out studies (Buerstedde et al., 2002). Although there have been extensive studies on important areas of cell biology using the DT40 cell system, there is only limited understanding of the miRNA expression and functions in this cell line. Global miRNA expression profiles of DT40 cells showed changes in the expression of a number of miRNAs (Figure 2). A number of miRNAs upregulated in DT40 cells also showed increased expression in CD40L-stimulated cells, suggesting that these miRNAs have a major role in cell proliferation. However, other miRNAs such as gga-miR-100 was up-regulated mainly in DT40 cells suggesting a more important role in transformation. Interestingly, miR-100 has been shown to be involved in a number of cancers in humans (Jung et al., 2011; Li et al., 2011; Oliveira et al., 2011; De Oliveira et al., 2012). On the other hand, gga-miR-146a, which was upregulated in CD40L stimulated B cells, is down-regulated in DT40 cells, providing further evidence for its role in the immune system.

A number of miRNAs down-regulated in DT40, including gga-miR-16, -30e, -30d, -30b, -30c, -26a, -147, -15b, and -29a, were also downregulated in CD40L-stimulated cells, suggesting conserved functions of cell division and proliferation. However, perhaps the most striking change is in gga-miR-155, which is the most up-regulated miRNA in CD40L stimulated B cells, but it is the most down-regulated miRNA in ALV transformed DT40 cells. One of the well characterized targets of miR-155, the transcription factor PU.1 is expressed in DT40 cells and has been shown to be important in the activation-induced cytidine deaminase (AID) expression and function (Luo and Tian, 2010). AID is important for B-cells to produce and maintain antibody diversity (Muramatsu et al., 2000). Reduced levels of miR-155 may help in maintaining high levels of PU.1, as these two have been shown to demonstrate inverse correlation in their expression (Thompson et al., 2011). Although it would be inaccurate to make direct comparisons of the miRNA profiles of DT40 cell lines and the naïve B cells stimulated with CD40L because of the significant differences between the two populations, the findings from the present study suggest the changes in the expression of some of these miRNAs could be contributing to the proliferative and unique recombinogenic properties of DT40 cells.

Macrophages play primary roles in both innate and adaptive immune responses, and *in vitro* studies on their function using macrophage cell lines have provided significant understanding on such responses. Avian myelocytomatosis virus (MC29)-transformed chicken macrophage cell line HD11 (Beug et al., 1979) is widely used in examining the innate immune functions. Although a number of miRNAs have been implicated in modulating innate immune functions, the miRNA profiles of these cells have not been examined. Although significant further studies are required to obtain the digital miRNA expression data of these cells, our data provide a snapshot of the expression profiles of the miRNAs that may have relevance to studies on their function. Among all the miRNAs expressed in these cells, gga-miR-21 alone accounted for nearly a third (28.8%). However, miR-21 is highly expressed in normal macrophages as well as the cESC line BP25. The data presented here could be valuable in examining the changes in miRNA expression profiles following the *in vitro* activation of specific signaling pathways, for which these cell types are widely used.

IAH30 is a turkey macrophage cell line (Lawson et al., 2001) transformed by the acutely transforming ALV subgroup J 966 virus with a transduced v-*myc* oncogene (Chesters et al., 2001). Although there has been progress in sequencing of the turkey genome (Dalloul et al., 2010), the annotation of the miRNAs is still not complete and no mature turkey miRNA sequences are available in miRBase. Hence we have used the deep sequencing data from IAH30 to examine the changes in the expression of turkey homologs of chicken miRNAs.

The two miRNAs that are significantly downregulated in IAH30 cells compared to HD11 –gga-miR-142-3p and gga-miR-223 (Figure 4B) have been shown to be involved in haemopoietic cell proliferation (Sun et al., 2010) and macrophage differentiation (Ismail et al., 2013). The vastly different abundances of these two key miRNAs suggest potential differences between the IAH30 and HD11 macrophage cell lines which may have implications for experiments which utilize them.

Analyzis of the miRNA profiles in the BP25 cESC line showed that the ES cell-specific miRNAs belonging to the miR-302-367 cluster were the most predominant miRNA population expressed in BP25. The expression levels of five miRNAs in the miR-302-367 cluster (miR-302a, miR-302b, miR-302c, miR-302d, and miR-367) accounted for 39.5% of all the sequenced miRNAs. This cluster of miRNAs is also expressed at high levels in human ESCs (Gunaratne, 2009), suggesting conserved functions of these miRNAs in mammals and birds. It has also been demonstrated in mammals that only a small subset of miRNAs, mostly seen as clusters in the genome, are expressed in the ES cells. For example, over 75% of the miRNAs expressed in mouse ES cells are represented by 6 loci (Calabrese et al., 2007). More recently, the miR-302-367 cluster was found to be highly expressed in differentiated blastoderm and primordial cells (Lee et al., 2011). Although shown to be significantly induced in human embryonal carcinoma cells (Suh et al., 2004), none of the miRNAs from the miR-302-367 cluster were detected in any of the other cell lines examined in this study, suggesting the suppression of these miRNAs upon differentiation. miRNAs belonging to the miR-302-367 cluster have been shown to regulate cell growth, metabolism, transcription (Dyce et al., 2010) and chromatin modification (Ren et al., 2009), demonstrating the potential importance of these miRNAs in maintaining the stem cell phenotype. BP25 cell line also showed high levels of expression of gga-miR-21 accounting for 23.3% of the miRNAome. Unlike in the BP25 cESC line, miR-21 levels are low in mammalian ES cells (Gunaratne, 2009), although the potential functional significance is not known. However, increased miR-21 expression is not unique to the chicken ES cell line, as high levels of expression of miR-21 have been detected in a number of human cancer cell lines (Slaby et al., 2007; Jiang et al., 2008). The miR-17-92 cluster of miRNAs (miR-17, miR-18a, miR-19a, miR-20a, miR-19b-1, and miR-92-1) was also expressed at relatively high levels (accounting for 12.4% of the miRNAome) in the BP25 cell line. As multifaceted miRNAs, these are not specific to the ESC, and have been associated with a number of cancers (Krichevsky and Gabriely, 2009), therefore the role of the miR-17-92 cluster in the BP25 cESC line may be related to its proliferative functions.

Taken together, we present the expression profiles of miRNAs determined by deep sequence analysis of the small RNA population from a number of avian cell types under conditions such as CD40L stimulation of B cells. The study also provides a snapshot of the miRNA profiles of cell lines such as DT40, HD11 and IAH30, transformed by the activation/transduction of *myc* oncogene. Although additional studies are required for precise characterization of the changes in miRNA expression and the functional significance of these changes, this study provides insights into the potential roles of miRNAs in the hematopoietic lineages of cells in a model non-mammalian species.

### Conflict of interest statement

The authors declare that the research was conducted in the absence of any commercial or financial relationships that could be construed as a potential conflict of interest.
